# Cardamom powder supplementation prevents obesity, improves glucose intolerance, inflammation and oxidative stress in liver of high carbohydrate high fat diet induced obese rats

**DOI:** 10.1186/s12944-017-0539-x

**Published:** 2017-08-14

**Authors:** Md Mizanur Rahman, Mohammad Nazmul Alam, Anayt Ulla, Farzana Akther Sumi, Nusrat Subhan, Trisha Khan, Bishwajit Sikder, Hemayet Hossain, Hasan Mahmud Reza, Md Ashraful Alam

**Affiliations:** 1 Department of Pharmaceutical Sciences, North South University, Dhaka, 1229 Bangladesh; 2 BCSIR Laboratories, Bangladesh Council of Scientific and Industrial Research (BCSIR), Dhaka, Bangladesh

**Keywords:** Cardamom, Glucose intolerance, Dyslipidemia, Obesity, Inflammation, Fibrosis

## Abstract

**Background:**

Cardamom is a well-known spice in Indian subcontinent, used in culinary and traditional medicine practices since ancient times. The current investigation was untaken to evaluate the potential benefit of cardamom powder supplementation in high carbohydrate high fat (HCHF) diet induced obese rats.

**Method:**

Male Wistar rats (28 rats) were divided into four different groups such as Control, Control + cardamom, HCHF, HCHF + cardamom. High carbohydrate and high fat (HCHF) diet was prepared in our laboratory. Oral glucose tolerance test, organs wet weight measurements and oxidative stress parameters analysis as well as liver marker enzymes such as alanine aminotransferase (ALT), aspartate aminotransferase (AST), alkaline phosphatase (ALP) activities were assayed on the tissues collected from the rats. Plasma lipids profiles were also measured in all groups of animals. Moreover, histological staining was also performed to evaluate inflammatory cells infiltration and fibrosis in liver.

**Results:**

The current investigation showed that, HCHF diet feeding in rats developed glucose intolerance and increased peritoneal fat deposition compared to control rats. Cardamom powder supplementation improved the glucose intolerance significantly (*p* > 0.05) and prevented the abdominal fat deposition in HCHF diet fed rats. HCHF diet feeding in rats also developed dyslipidemia, increased fat deposition and inflammation in liver compared to control rats. Cardamom powder supplementation significantly prevented the rise of lipid parameters (*p* > 0.05) in HCHF diet fed rats. Histological assessments confirmed that HCHF diet increased the fat deposition and inflammatory cells infiltration in liver which was normalized by cardamom powder supplementation in HCHF diet fed rats. Furthermore, HCHF diet increased lipid peroxidation, decreased antioxidant enzymes activities and increased advanced protein oxidation product level significantly (*p* > 0.05) both in plasma and liver tissue which were modulated by cardamom powder supplementation in HCHF diet fed rats. HCHF diet feeding in rats also increased the ALT, AST and ALP enzyme activities in plasma which were also normalized by cardamom powder supplementation in HCHF diet fed rats. Moreover, cardamom powder supplementation ameliorated the fibrosis in liver of HCHF diet fed rats.

**Conclusion:**

This study suggests that, cardamom powder supplementation can prevent dyslipidemia, oxidative stress and hepatic damage in HCHF diet fed rats.

## Background

Now a day, obesity is a notable life threatening disease which is associated with the prevalence of metabolic syndrome including central obesity, insulin resistance, impaired glucose tolerance, diabetes, hypertension, and dyslipidemia [1, 2]. According to various studies, the most common causes of obesity is excess dietary energy intake [3]. Other causes of this metabolic imbalance are western style diet, high fat and sugar, together with a sedentary lifestyle. When energy intake exceeds energy expenditure, excessive cellular lipid accumulation occurs not only in adipose tissue but also in ectopic tissues such as liver [4]. Excessive ectopic lipid deposition often disrupts normal cellular and physiological function, which if allowed to proceed unchecked, will lead to pathological progression [5]. With the progress of obesity, increased hepatic lipogenesis and serum fatty acid lead to excess accumulation of liver lipid which result in fatty liver, impaired liver function, and eventually liver failure [6].

Plant phenolics showed proven lipid lowering effect in experimental animal and clinical setup which are recently reviewed by Scicchitano et al. [7]. An important nutraceutical antioxidants quercetin showed reduction of oxidative stress, maintained blood glutathione redox ratio, preserved vascular function in male ICR mice [8]. Another antioxidant compound resveratrol which can be found in grape seeds, showed lipid lowering effect and lowered TC and LDL-C and increased HDL-C than HFD group in Male C57BL/6 J mice [9]. Resveratrol treatment also reduced the hepatic cholesterol accumulation than HFD group [9].

Likewise, plant sterols, grape seed proanthocyanidine, anthocyanins, soy protein and probiotics also showed beneficial lipid lowering effects [7].

Generally, spices are used to increase the flavor of foods. But spices may also be useful as medicinal agents which can enhance the immune system of our body and decrease the chances of occurring various life threatening diseases. In particular, spices may decrease metabolic syndrome, obesity, hypertension, diabetes and non-alcoholic fatty liver disease [10, 11]. Cardamom (*Elettaria cardamomum* Maton (family; *Scitaminaceae*)) is a perennial herb, indigenous to India, Pakistan, Burma and Bangladesh, locally known as “elaichi”. It is also used in culinary and traditional medicine practices. Cardamom is a good source of volatile oils, fixed oils, phenolic acids and sterols [12]. Phytochemical studies revealed the presence of multiple chemicals, such as α-terpineol, myrcene, heptane, subinene, limonene, cineol, α-phellandrene, menthone, α-pinene, β-pinene, β-sitostenone, γ-sitosterol, phytol, eugenyl acetate [13]. In folkloric medicine, cardamom is used as carminative, stomachic, diuretic, antibacterial, antiviral, antifungal and is considered useful in treatment of constipation, colic, diarrhea, dyspepsia, vomiting, headache, epilepsy and cardiovascular diseases [14]. Volatile oils in cardamom was found to exhibit analgesic, anti-inflammatory, antimicrobial and antispasmodic properties [15, 16]. Moreover, cardamom fruit is used against cardiac disorders, renal and vesicular calculi, dyspepsia, debility, anorexia, asthma, bronchitis, halitosis and gastrointestinal disorders [17]. Cardamom also possesses antioxidant, antihypertensive, gastro protective, and antispasmodic, antibacterial, antiplatelet aggregation and anticancer properties [18]. Considering all above reports, the current investigation was undertaken to evaluate the potential benefit of cardamom powder supplementation in high carbohydrate high fat (HCHF) diet induced obese rats.

## Methods

### Chemicals

The beef tallow used as high fat source was obtained from the local beef market and processed well by heating to solidify it for using in HCHF formulation. Thiobarbituric acid (TBA) was purchased from Sigma Chemical Company (USA). Reduced glutathione (GSH) was obtained from J.I. Baker (USA). Alanine aminotransferase (ALT), aspartate aminotransferase (AST), alkaline phosphatase (ALP), Triglyceride liquid, Cholesterol (total) liquid and LDL assay kits were purchased from DCI diagnostics (Budapest, Hungary). All other chemicals and reagents used were of analytical grade.

### Plant material

Cardamom was collected from the local market of Dhaka, Bangladesh. Then it was grinded in a grinder machine to get coarse powder to be used as supplementation with diet.

### HPLC detection and quantification of polyphenolic compounds

Detection and quantification of selected phenolic compounds in the ethanol extract were determined by HPLC-DAD analysis as described by [19]. It was carried out on a Dionex UltiMate 3000 system equipped with quaternary rapid separation pump (LPG-3400RS) and photodiode array detector (DAD-3000RS). Separation was performed using Acclaim® C_18_ (5 μm) Dionex column (4.6 × 250 mm) at 30 °C with a flow rate of 1 ml/min and an injection volume of 20 μl. The mobile phase consisted of acetonitrile (solvent A), acetic acid solution pH 3.0 (solvent B), and methanol (solvent C) with the gradient elution program of 5%A/95%B (0-5 min), 10%A/90%B (6-9), 15%A/75%B/10%C (11-15), 20%A/65%B/15%C (16-19 min), 30%A/50%B/20%C (20-29 min), 40%A/30%B/30%C (30-35) and 100%A (36-40 min). The UV detector was set to 280 nm for 22.0 min, changed to 320 nm for 28.0 min, again change to 280 nm for 35 min and finally to 380 nm for 36 min and held for the rest of the analysis period while the diode array detector was set at an acquisition range from 200 nm to 700 nm. For the preparation of calibration curve, a standard stock solution was prepared in methanol containing arbutin (AR), (−)-epicatechin (ECA) (5 μg/ml each), gallic acid (GA), hydroquinone (HQ), vanillic acid (VA), rosmarinic acid (RA), myricetin (MC) (4 μg/ml each), caffeic acid (CA), Syringic acid (SA), vanillin (VL), trans-ferulic acid (FA) (3 μg/ml each), *p*-coumaric acid (PCA), quercetin (QU), kaempferol (KF) (2 μg/ml each), (+)-catechin hydrate (CH), ellagic acid (EA) (10 μg/ml each), trans-cinnamic acid (TCA) (1 μg/ml), rutin hydrate (RH) (6 μg/ml) and benzoic acid (BA) (8 μg/ml). A solution of the extract was prepared in ethanol having the concentration of 10 mg/ml. Prior to HPLC analysis, all the solutions (mixed standards, sample and spiked solutions) were filtered through 0.20 μm syringe filter (Sartorius, Germany) and then degassed in an ultrasonic bath (Hwashin, Korea) for 15 min. Data acquisition, peak integration and calibrations were calculated with Dionex Chromeleon software (Version 6.80 RS 10).

### Animals and treatment

All experimental protocols were approved by the Ethical Committee of North South University for animal care and experimentation. The experimental group consisting of 28 Wistar male rats (10 to 12 weeks old, 185-200 g) were obtained from Animal production unit of Animal House at Department of Pharmaceutical Sciences, North South University and kept in individual cages at temperature controlled room (22 ± 3 °C), humidity 55%with a 12 h dark/light cycles environment having free access to standard laboratory feed and water. To study the effects of high carbohydrate high fat diet and its attenuation by supplementation of cardamom, rats were randomly divided into four experimental groups (*n* = 7 each), such as

- Control (group I), received normal water and chow food (crashed as powder) for 8 weeks.
- Control + Cardamom (group II), received normal water and chow food (crashed as powder) with cardamom (1% of powder chow diet, *w*/w) for 8 weeks.
- HCHF (Group III), HCHF diet for 8 weeks.
- HCHF+ Cardamom (Group IV). HCHF diet with cardamom (1% of HCHF diet, *w*/w) for 8 weeks.

HCHF diet was prepared in our laboratory in pellet forms (Table 1). To assess the glycemic activity before and after the HCHF feeding, OGTT was performed for all four groups before and after finishing of treatment. Measurements of body weight, food and water intakes were recorded daily. After 56 days, all animals were weighed and sacrificed by using high dose of pentobarbital anaesthesia (90 mg/kg) injection in peritoneal region. The blood was collected from abdominal aorta and placed into heparinized tubes at 4 °C. Blood samples were centrifuged at 8000 rpm, 4 °C for 15 min within 30 min of collection to separate the plasma. Separated plasma was transferred to eppendorf tubes and stored at −20 °C for further analysis.

Moreover, all other internal organs like adipose tissues, heart, kidney, spleen and liver were also immediately collected from euthanized animals; they were also weighed and stored in neutral buffered formalin (pH 7.4) for histological analysis and in refrigerator at −20 °C for further biochemical studies.

### Oral glucose tolerance test

At the end of the feeding protocol, rats were kept starved overnight (12 h) and an oral glucose tolerance test was performed. Normal water was supplied during the food deprivation period. Basal blood glucose concentrations were measured in blood taken from the tail vein using glucometer (Bionim Corporation, Bedford, MA, USA). The rats were administered 2 g/kg body weight of glucose as a 40% aqueous solution via oral gavage. Tail vein blood samples were taken at 30, 60, 90 and 120 min following glucose administration.

### Plasma biochemistry

Blood was centrifuged at 8000 rpm for 15 min within 30 min of collection into heparinized tubes. Plasma was separated and transferred to Eppendorf tubes for storage at −20°c before analysis. Plasma concentrations of total cholesterol, triglycerides, LDL, HDL and activities of plasma alanine transaminase (ALT), aspartate transaminase (AST) and alkaline phosphatase (ALP) were determined using kits supplied by Diatec diagnostic kits (Hungary) according to manufacturer-provided standards and protocols. Plasma insulin was estimated using Diatec diagnostic kits (Hungary) according to the manufacturer’s protocol.

### Preparation of tissue samples and assay of oxidative stress markers

For determination of oxidative stress markers, liver tissue was homogenized in 10 volumes of Phosphate buffer containing (pH 7.4) and centrifuged at 8000 rpm for 15 min at 4 °C. The supernatant was collected and used for the determination of protein and enzymatic studies as described below.

Lipid peroxidation in liver was estimated calorimetrically measuring thiobarbituric acid reactive substances (TBARS) followed by previously described method [20]. The absorbance of clear supernatant was measured against reference blank at 535 nm. Nitric oxide (NO) was determined according to the method described by Tracey et al. as nitrate [21]. The absorbance of the final solution was measured at 540 nm against the corresponding blank solutions. NO level was measure by using standard curve and expressed as nmol/g of tissue. Determination of APOP levels was performed by modification of the method of Witko-Sarsat et al. [22] and Tiwari et al. [23]. The chloramine-T 7 absorbance at 340 nm was found linear within the range of 0 to 100 nmol/mL, AOPP concentrations were expressed as nmol·mL − 1 chloramine-T equivalents.

### Antioxidant enzyme activities such as catalase activity assay (CAT), superoxide dismutase (SOD) activity assay and reduced glutathione assay (GSH)

CAT activities were determined using previously described method by Chance and Maehly [24] with some modifications. Changes in absorbance of the reaction solution at 240 nm were determined after 1 min. One unit of CAT activity was defined as an absorbance change of 0.01 as units/min. SOD was assayed in plasma and tissue homogenates by using previously described method [25]. Changes in absorbance were recorded at 480 nm for 1 min at 15 s interval. Control consisting of all the ingredients, except enzyme preparation, was run simultaneously. One unit of enzyme activity has been defined to cause 50% inhibition of auto-oxidation of epinephrine present in the assay system. Reduced glutathione was estimated by the method of Jollow et al. [26]. The yellow color of the mixture was developed, read immediately at 412 nm on a Smart SpecTM plus Spectrophotometer and expressed as ng/mg protein.

### Estimation of myeloperoxidase (MPO) activity

MPO activity was determined by a dianisidine-H_2_O_2_method [27], modified for 96-well plates. Briefly, plasma samples (10 μg protein) were added in triplicate to 0.53 mM *o*-dianisidine dihydrochloride (Sigma) and 0.15 mM H_2_O_2_ in 50 mM potassium phosphate buffer (pH 6.0). The change in absorbance was measured at 460 nm. Results were expressed as units of MPO/mg protein.

### Histopathalogical determination

For microscopic evaluation liver tissues were fixed in neutral buffered formalin and embedded in paraffin, sectioned at 5 μm and subsequently stained with hematoxylin/eosin to see the architecture of hepatic tissue and inflammatory cell infiltration. Sirius red staining for fibrosis and Prussian blue staining for iron deposition were also done in liver sections. Sections were then studied and photographed under light microscope (Zeiss Axioscope) at 40X magnifications.

### Statistical analysis

All values are expressed as mean ± standard error of mean (SEM). The results were evaluated by using the One-way ANOVA followed by Newman- Keuls post hoc test using Graph Pad Prism Software. Statistical significance was considered at *p* < 0.05 in all cases.

## Results

### Analysis of ethanol extract of cardamom by HPLC-DAD

**Fig. 1:**
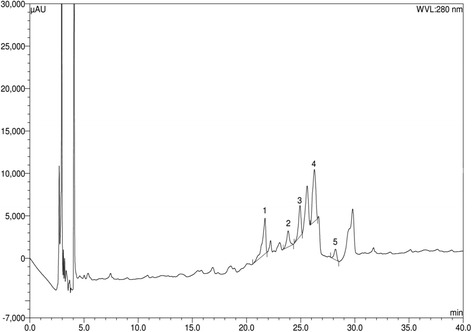
HPLC chromatogram of ethanol extract of Cardamom Peaks: 1, (−)-epicatechin (ECA); 2, vanillin (VL); 3, *p*-coumaric acid (PCA); 4, *trans*-ferulic acid (FA); 5, ellagic acid (EA)

Identification and quantification of individual polyphenolic compounds in the ethanol extract of Cardamom were analysed by HPLC. The chromatographic separations of polyphenols in ethanol extract are shown in Fig. 1. The content of each phenolic compound was calculated from the corresponding calibration curve and presented as the mean of five determinations as shown in Tables 1 and 2. According to our HPLC analysis, we found that ethanol extract of cardamom consists of (−)-epicatechin, vanillin, *p*-coumaric acid, trans-ferulic acid, ellagic acid. This HPLC procedure provided excellent identification and quantification of these phenolic compounds presented in the ethanol extract of Cardamom within a short analysis time (40 min).

**Table 1:** Composition of normal and high carbohydrate high fat diet used in this study (for 100 g)

Ingredients of Normal lab diet	%	Ingredients of HCHF diet	%
Wheat	40%	Powdered normal rat feed	15.5%
Wheat bran	20%	Sugar	17.5%
Rice Polishing	5.5%	Beef tallow (fat)	20.0%
Fish meal	10.0%	Condensed milk	39.5%
Oil cake	6.0%	Vit-B complex	0.1%
Gram	0.39%	Salt	0.5%
Pulses	0.39%	Water	100 ml
Milk	0.38%		
Soybean Oil	1.5%		
Molasses	0.095%		
Salt	0.095%		
Embavit (vitamin)	0.1%		

Normal chow diet contained as percentage of calories 14% proteins, 57% carbohydrates, 13.5% fat. High Carbohydrate high fat diets contained as percentage of calories 14% proteins, 37% carbohydrates, 48% fat

**Table 2:** Contents of polyphenolic compounds in the ethanol extract of cardamom (*n* = 5)

Polyphenolic compound	Ethanol extract of cardamom	
	Content (mg/100 g of dry extract)	% RSD
ECA	56.28	1.39
VL	2.17	0.05
PCA	4.06	0.08
FA	14.93	0.46
EA	9.88	0.12

ECA, (−) epicatechin; VL, vanillin; PCA, *p-*coumaric acid; FA, Ferulic acid; EA, Ellagic acid

### Effect on body weight, food and water intake

**Fig. 2:**
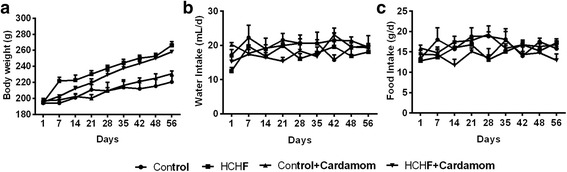
Effect of Cardamom Powder supplementation on body weight (**a**), food (**b**) and water (**c**) intake in high fat diet induced obese rats. Values are presented as mean ± SEM. *n* = 7

The body weight, food intake and water intake of each rat was noted every day during the experimental period. In the Fig. 2 and Table 3, the change of body weight, food intake and water intake at the time of study are shown. Significant body weight gain was found in HCHF rats compared to the control rats (Fig. 2a). Cardamom powder supplementation showed decreased body weight gain in comparison to HCHF diet fed rats but the changes were not significant. No significant difference of food and water intake were also observed among the four groups of animals tested in this study (Fig. 2b and c).

**Table 3:** Effects of HCHF diet on body weight, food and water intake and organ weight

Parameters	Control	Control + Cardamom	HCHF	HCHFF+ Cardamom
Initial body weight (g)	194.25 ± 1.68	197.23 ± 2.95	195.18 ± 1.31	195.55 ± 3.39
Final body weight (g)	220.68 ± 7.14	230.83 ± 3.65	266.40±4.33	258.15 ± 1.58
Food intake /d (g)	14.60 ± 0.70	15.92 ± 0.74	14.81 ± 0.28	12.93 ± 1.55
Water intake/ d(g)	19.53 ± 0.85	18.14 ± 1.71	15.00 ± 1.00	15.76 ± 0.78
Calorie Intake (kj/day)	307.6 ± 7.3a	305.1 ± 3.5a	412.9 ± 7.5b	385.3 ± 6.0b,c
Liver wet weight (g/ 100 g of body weight)	3.51 ± 0.05	2.74 ± 0.23	3.63 ± 0.09	2.70 ± 0.06
Heart wet weight (g/ 100 g of body weight)	0.28 ± 0.01	0.26 ± .00	0.30 ± 0.01	0.27 ± 0.01
Pancreas wet weight (g/ 100 g of body weight)	0.32 ± 0.01	0.37 ± 0.02	0.34 ± 0.02	0.34 ± 0.03
Fat deposition				
Paritoneal fat (g/ 100 g of body weight)	1.12 ± 0.12a	0.91 ± 0.08a	2.37 ± 0.16b	1.65 ± 0.15a
Epididymal fat (g/ 100 g of body weight)	0.91 ± 0.08	1.05 ± 0.15	1.14 ± 0.09	1.06 ± 0.09
Mesenteric (g/ 100 g of body weight)	0.69 ± 0.19	0.45 ± 0.07	0.63 ± 0.09	0.83 ± 0.07

Values are presented as mean ± SEM. *n* = 7. One way ANOVA followed by Newman-Keuls post hoc test were done for statistical comparison. Values are considered significance at *p* < 0.05. a vs b, control vs HCHF or HCHF vs tretment which are different at *p* < 0.05

### Effect of cardamom powder on oral glucose tolerance test

**Fig. 3:**
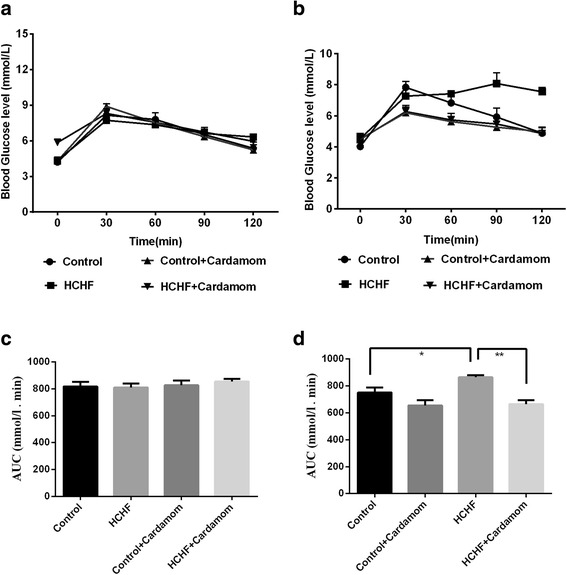
Effect of Cardamom powder supplementation on body weight, oral glucose tolerance test (OGTT) before and after the high fat high carbohydrate diet feeding in rats. Values are presented as mean ± SEM, *n* = 7. One way ANOVA followed by Newman-Keuls post hoc test were done for statistical comparison. Values are considered significance at *p* < 0.05. **a** OGTT before tretment. **b** OGTT after tretment. **c** OGTT AUC BF. **d** OGTT AUC AF

The results of oral glucose tolerance test (OGTT) of the control and experimental obese rats are shown in Fig. 3. OGTT test in rats at the beginning of the study did not produced any significant changes among the groups tested (Fig. 3a). However, post treatment OGTT test revealed that, in normal control rats, maximum elevation in blood glucose level was observed at 60 min after glucose load and declined to near basal level at 120 min, whereas, in HCHF-induced obese rats, the peak increase in blood glucose level was noticed even after 60 min and remained high over the next 60 min (Fig. 3b). Interestingly, supplementation of cardamom powder to obese rats elicited a significant decrease in blood glucose level at 60 min and beyond when compared with HCHF diet fed rats (Fig. 3b). The AUC of OGTT at the beginning of the study did not show any changes among the groups studied (Fig. 3c). The AUC of OGTT at the end of the study in HCHF diet fed rats significantly increased compared to control rats which was further normalized by cardamom treatment (Fig. 3d).

### Effect on organ wet weight

In the Table **3** **,** the effect of various treatments on the rats organ wet weights are shown. In comparison to the control group, the liver wet weight was not changed significantly in the HCHF diet fed rats. Cardamom powder (1% *w*/w of diet) supplementation, however, significantly (*p* < 0.05) decreased the wet weight of the liver in the HCHF-diet fed rats. HCHF diet fed rats also showed no changes in heart and pancreas wet weights compared to control rats; however, **c**ardamom powder supplementation did not affect any of these wet weights in rats.

### Effect of cardamom powder supplementation on accumulations of fat

A very interesting result was observed in the case of fat accumulation on feeding of HCHF diet particularly in HCHF diet fed rats. Vivid differences in fat pad deposition levels were observed in terms of peritoneal fat tissues (Table 3). The wet weights of peritoneal, adipose fat pads were markedly increased in HCHF-fed groups compared to control rats. Cardamom powder supplementation reduced the wet weight of peritoneal fat pad wet weights considerably (Table 3). HCHF diet or cardamom powder supplementation showed no effect on mesenteric and epididymal fat deposition among the groups studied (Table 3).

### Effect of cardamom powder supplementation on cholesterol and triglyceride level

Table 4 shows the plasma concentration of cholesterol and triglyceride in all groups of rats. Cholesterol and triglyceride concentrations were significantly increased in HCHF diet fed rats compare to control group. Cardamom powder supplementation signifiantly reduced cholesterol and triglyceride concentration in HCHF diet fed rats. Moreover, LDL cholestrarol level was also increased in HCHF diet fed animals compared to control rats, which was further lowered by cardamom powder supplementation (Table 4). However, HDL concentrations were increased in HCHF and control diet fed groups supplemented with cardamom powder (Table 4). Cardamom powder supplementation in control rats did not alter any other (Cholesterol, tryglyceride and LDL) lipid parameters in this study.

**Table 4:** Effects of fat diets on biochemical parameters in plasma and liver

	Control	Control + Cardamom	HCHF	HCHFF+ Cardamom
Plasma				
AST(U/L)	28.71 ± 3.63a	22.97 ± 3.63a	57.42 ± 6.58b	30.15 ± 1.93a
ALT(U/L)	34.45 ± 4.45a	35.89 ± 2.65a	57.42 ± 1.82b	40.20 ± 3.63a
ALP(U/L)	60.67 ± 3.36a	50.38 ± 5.38a	102.19 ± 5.69b	47.49 ± 1.46a
MDA(nmol/mL)	29.10 ± 2.06a	27.61 ± 2.49a	54.43 ± 2.12b	20.48 ± 1.14a
NO(nmol/mL)	8.44 ± 0.66a	9.77 ± 1.28a	17.36 ± 1.34b	8.87 ± 0.47a
APOP	268.81 ± 23.38a	185.08 ± 14.99a	570.40 ± 42.42b	148.17 ± 11.25a
Catalase(U/min)	19.67 ± 3.41a	16.17 ± 1.68a	8.83 ± 0.87b	14.50 ± 1.80a
GSH (μg/mg protein)	23.33 ± 1.74a	29.01 ± 1.40a	14.40 ± 0.38b	26.02 ± 0.58a
SOD (U/L)	43.47 ± 3.55a	51.86 ± 8.19a	15.34 ± 2.33b	44.32 ± 7.73a
Cholesterol(mg/dL)	155.18 ± 8.31a	163.03 ± 4.04a	290.25 ± 19.47b	183.70 ± 5.24a
Triglycerides(mg/dL)	196.75 ± 8.31a	160.3 ± 5.1a	316.89 ± 22.31b	169.32 ± 13.44a
HDL (mg/dL)	41.14 ± 0.97a	66.66 ± 4.51b,c	54.56 ± 2.79b	66.44 ± 3.05b,c
LDL (mg/dL)	74.69 ± 8.12a	64.30 ± 4.64a	172.31 ± 20.35b	83.40 ± 3.97a
Liver				
MDA(nmol/g tissue)	89.41 ± 4.73a	101.85 ± 7.17a	146.21 ± 8.82b	117.49 ± 8.16a
NO(nmol/g tissue)	25.68 ± 1.98a	32.79 ± 5.51a	84.87 ± 10.8b	40.91 ± 3.63a
APOP	583.65 ± 51.80a	489.21 ± 20.88a	1384.44 ± 84.44b	420.95 ± 82.05a
Catalase(U/min)	25.00 ± 4.28a	20.00 ± 2.58a	10.83 ± 1.54b	22.50 ± 4.43a
GSH(μg/mg protien)	29.48 ± 2.37a	29.01 ± 1.40a	11.09 ± 0.40b	26.02 ± 0.58a
SOD (U/g tissue)	42.95 ± 6.74a	43.85 ± 4.21a	17.71 ± 1.80b	41.90 ± 6.44a
MPO (U/ g tissue)	0.35 ± 0.06a	0.44 ± 0.05a	1.37 ± 0.08b	0.93 ± 0.16a

Values are presented as mean ± SEM. *n* = 7. One way ANOVA followed by Newman-Keuls post hoc test were done for statistical comparison. Values are considered significance at *p* < 0.05. a vs b, control vs HCHF or HCHF vs tretment which are different at *p* < 0.05

### Serum ALT, AST and ALP activities

HCHF diet feeding in rat significantly increased the liver marker enzymes such as ALP, ALT and AST activities compared to the control rats. Supplementation with cardamom powder (1% cardamon) significantly decreases the ALP, ALT and AST enzymatic activities and ameliorates the function of liver. ALP, ALT and AST enzymatic activities are given in Table 4.

### Evaluation of plasma and liver oxidative stress markers

To assess the oxidative stress condition, MDA, NO and APOP concentrations were measured in plasma and liver. Lipid peroxidation was evaluated by measuring malondialdehyde (MDA) formation. MDA concentration in plasma and liver was significantly elevated in HCHF treated rats compared to the control rats (Table 4). On the contrary, Cardamom supplementation (1% cardamon) in HCHF diet fed rats significantly (*p* < 0.05) reduces the MDA concentration in plasma and liver. Advanced protein oxidation products (APOP) was also increased in HCHF diet fed rats compared to control. Supplementation with cardamom in rats exhibited significant decreases in APOP concentration in both plasma and liver (Table 4). Nitric oxide was also measured for the determination of oxidative stress in plasma and liver. Level of nitric oxide in HCHF diet fed rats was increased whereas decreased level was exhibited by the cardamom supplementation in rats.

### Antioxidant enzymes and glutathione status

Catalase, SOD and GSH are cellular antioxidants which reduce the oxidative stress. Activity of catalase was significantly depleted in HCHF diet fed rats compared to control whereas catalase activity was significantly increased in rats supplemented with cardamom (1% cardamon) in both plasma and liver*.* Another antioxidant enzyme is SOD (Superoxide dismutase) which also showed significant depletion in HCHF diet fed rats compared to the control rats. Cardamom supplementation in rats restores the level of SOD significantly and improves the condition of plasma and liver (Table 4). Glutathione (GSH) concentration was also depleted in both liver and plasma of HCHF diet fed rats compared to the control rats (Table 4). On the other hand, cardamom supplementation in rats demonstrated significant increase in the level of GSH in liver.

### Effect of cardamom powder on hepatic inflammatory cells infiltration and fibrosis in HCHF diet rats

**Fig. 4:**
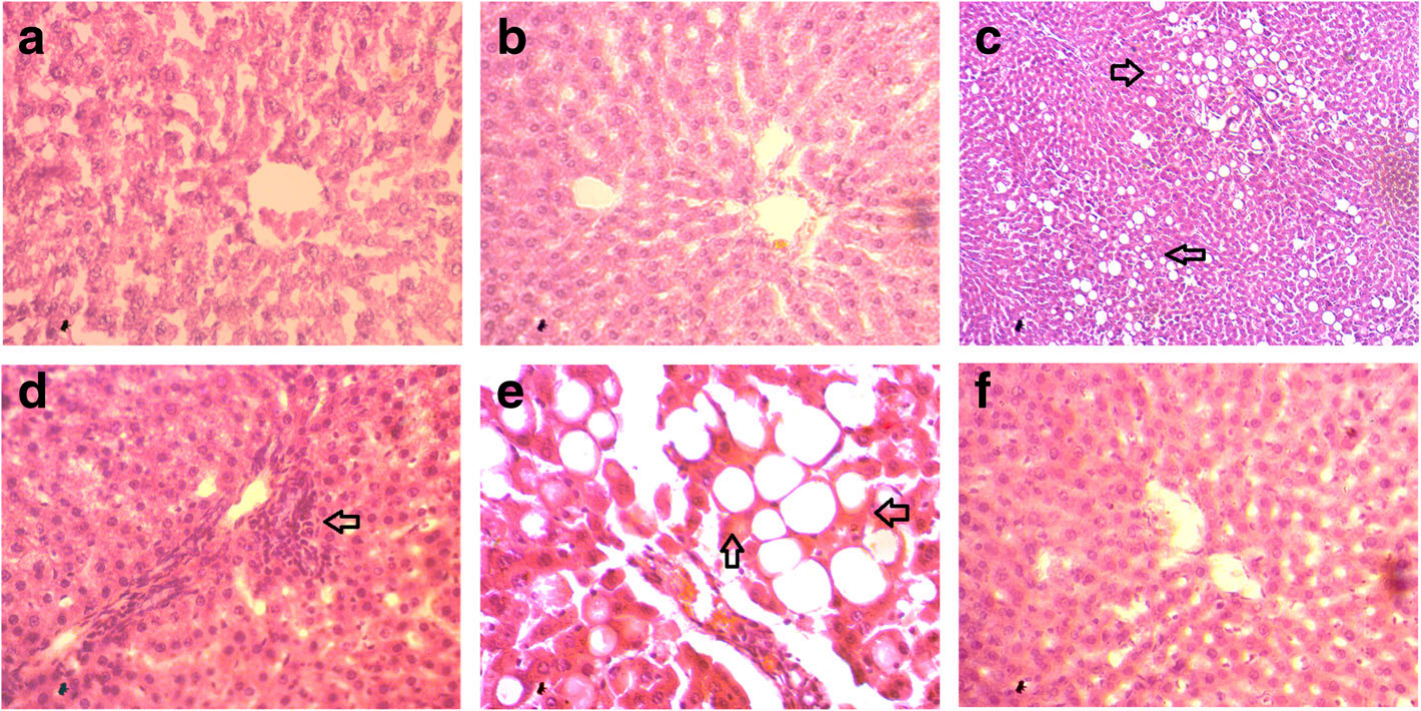
Effect of cardamom powder on hepatic inflammatory cells infiltration in HCHF diet rats. **a** Control, (**b**) Control + Cardamom; (**c**) HCHF (10× magnification); (**d**) HCHF, inflammatory cells infiltration, (**e**) HCHF (fat droplet deposition) and (**f**) HCHF + Cardamom. Magnifications 40X

**Fig. 5:**
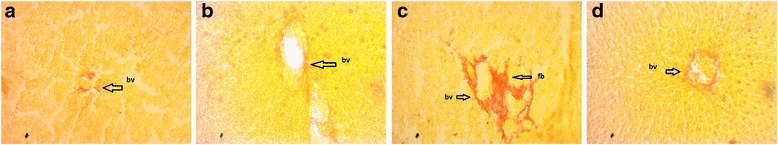
Effect of cardamom powder on hepatic fibrosis in HCHF diet rats. a Control, (b) Control + Cardamom; (c) HCHF; (d) HCHF + Cardamom. Magnifications 40X

**Fig. 6:**
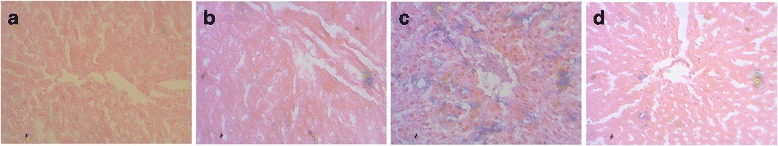
Effect of cardamom powder on hepatic iron deposition (*blue* deposits) in HCHF diet rats. **a** Control, (**b**) Control + Cardamom; (**c**) HCHF; (**d**) HCHF + Cardamom. Magnifications 40X

H and E staining analysis of the liver of control and cardamom supplemented control diet fed rats showed normal architecture of hepatocytes (Fig. 4a and b). However, the liver of HCHF diet feed rats showed marked vacuolar degeneration indicating hepatic fat accumulation (Fig. 4c and e). The HCHF diet fed rats also showed greater infiltration by inflammatory cells (Fig. 4d) as well as increased interstitial collagen deposition in liver (Fig. 5c) compared to Control (Fig. 5a) rats. Cardamom supplementation (1% of powder chow diet) markedly reduced inflammation (Fig. 4f) and collagen deposition (Fig. 5d) in HCHF diet fed rats whereas Cardamom supplementation in control rats did not show any alteration compared to control rats. Moreover, free iron deposition was found in liver section of HCHF diet fed rats compared to control rats (Fig. 6c) which was further ameliorated by cardamom powder supplementation (Fig. 6d).

## Discussion

Obesity is a global health concern because of the ever increasing rates over the last three decades worldwide. Obesity is also related with nonalcoholic fatty liver disease (NAFLD). It increases the triglyceride content in human body that causes steatosis, associated with inflammation and fibrosis i.e. steatohepatitis. Moreover, the presence of NAFLD is associated with a high risk of developing type 2 diabetes mellitus, hypertension as well as dyslipidemia [28, 29]. In this current study, high carbohydrate high fat (HCHF) diet fed rats showed development of metabolic syndrome, hyperlipidemia, glucose intolerance and central obesity which were further ameliorated by cardamom powder supplementation.

This study also showed that high fat diet feeding in rats developed glucose intolerance compared to the control rats. This finding is in agreement with previously published work showed that HCHF diet fed rats develops glucose intolerance and insulin resistance [30, 31]. Several mechanisms have been suggested for the development of glucose intolerance and insulin resistance in high fat diet induced obesity. Oxidative stress in adipose tissue is one of the early events in the development of metabolic syndrome in obesity [32]. Oxidative stress is known to impair both insulin secretion by pancreatic β cells, and glucose transport in muscle and adipose tissue [33–35]. Our investigation also revealed that HCHF diet feeding in rats increased the oxidative stress parameters both in plasma and tissues followed by a decrease in cellular antioxidant capacities. Moreover, cardamom supplementation in HCHF diet fed rats improves the glucose metabolism and prevented the rise of oxidative stresses. These findings are also supported by previously published works showed that cardamom improved hyperglycemia, glucose intolerance and insulin resistance in rats [36, 37].

HCHF diet induced hyperlipidemia in the animals, as confirmed by the higher serum lipid profile compared with rats fed a control chow diet [38]. This study revealed that cardamom supplementation was able to reduce the cholesterol which was increased by HCHF diet. Previous report suggests that cardamom can prevent the rise of cholesterol level in plasma due to high fat diet feeding in rats [36]. The increase in cholesterol may potentially enhance the risk of fatty liver diseases [39]. In this study, HCHF diet feeding in rats increased the fat accumulation and increased lipid peroxidation in liver of rats and raised the liver wet weight. Moreover, increased activities of plasma ALT, AST and ALP enzymes were also observed in HCHF diet fed rats. Oxidative stress mediated tissue damage in liver may increase the liver markers enzyme activities in plasma. It mainly happens when there is any damage or deterioration in liver, damage to the liver increase the leaking of ALT and AST into the blood stream. Cardamom improved liver function since it normalized the liver wet weight and also normalize plasma activities of ALT, AST and ALP [40]. Similarly, improved hepatic function was measured with cardamom extract in alcohol-induced liver damage [41].

Oxidative stress is a major cause of different complications originating from obesity [42]. It is found that concentration of MDA, NO and APOP which are indices of oxidative stress were significantly elevated in liver of HCHF administered rats, and treatment with cardamom reduces these marker compare with HCHF rats. The level of antioxidant enzymes like catalase, and SOD activities and GSH concentration in the liver of HCHF diet fed rats were seen significantly lower than control group which signifies that obesity has reduced the antioxidant capacity of the liver cells [35, 43]. Cardamom supplementation restored these enzymes activities in HCHF diet fed rats, which indicates that cardamom improve the antioxidant capacity by increasing antioxidant enzymes.

HCHF diet induced liver dysfunction in this model is associated with oxidative stress, inflammatory cells infiltration and fibrosis as seen in biochemical and histological examinations. Day and colleagues presented an important theoretical framework more than a decade ago, termed the “two-hit hypothesis,” which suggests that after an initial hit (i.e., hepatic steatosis), the second hit (i.e., inflammation) is needed to develop non-alcoholic fatty liver diseases [44]. Hepatocytes possess an integrated system of enzymatic and non-enzymatic antioxidant defenses to remove or neutralize ROS. However, sudden increase in saturated fatty acid inflow in the liver increased the ROS production and destabilized the antioxidant defense mechanism as discussed before [45, 46]. It is now evident that ROS may stimulate hepatic stellate cells and emphasize the production of type I collagen, therefore increasing extracellular matrix deposition during fibrogenesis [47]. Moreover, the local macrophages which are also known as Kupffer cells can recruit monocytes in the injured area of liver and have cross talk between HSCs [48, 49]. In this study, high fat diet feeding in rats showed massive recruitment of inflammatory cells in the liver followed by increased in extracellular matrix deposition supports the notion of hepatic injury. Cardamom supplementation prevented the recruitment of inflammatory cells and extracellular matrix deposition in the liver of HCHF diet fed rats. The protective effect is mediated due to the restoration of antioxidant capacity and lipid lowering effect of cardamom supplementation [36].

In this study cardamom is used to prevent obesity related metabolic syndrome. Cardamom contains good amounts of phenolic and flavonoid components that may have biological activity [50]. HPLC analysis showed that ethanol extract of cardamom consists of (−)-epicatechin, vanillin, *p*-coumaric acid, trans-ferulic acid, ellagic acid which have highly anti inflamatory and antioxidant activities. According to the literature review of cardamom, the major constituents of cardamom are α-terpinyl acetate, α-terpineol, 1,8-cineole and limonene, which have potential effects in metabolic syndrome as these terpenes reduced blood pressure in normotensive rats and also showed endothelium dependent vasorelaxation in male Wistar rats [12]. However, this previous report ignored the potential role of phenolic antioxidants present in cardamom. Our report suggests that a synergistic effect could be possible due to the presence of wide range of bioactive components present in cardamom powder.

## Conclusion

The current investigation showed beneficial role of cardamom powder supplementation in high carbohydrate high fat (HCHF) diet induced obese rats. Cardamom powder supplementation lowered the rise of total cholesterol and triglyceride level as well as peritoneal fat deposition in HCHF diet induced obese rats. Cardamom powder supplementation also prevented the hepatic dysfunction as shown by lowering AST, ALT and ALP enzyme activities. The presence of phenolic compounds in cardamom powder may be responsible for the hepatic protection and improved anti-oxidant capabilities in HCHF diet induced obese rats. However, volatile oil principle present in cardamom cannot be ignored for the said activities. Investigating with the individual phenolic compounds and volatile terpenes in HCHF diet induced obese rats would address their role in preventing metabolic syndrome in this animal model, which could not be addressed in this study. However, this study opens a new avenue of research for functional food concept using cardamom powder in ameliorating obesity and metabolic syndrome. Further studies are warranted to find out the benefit in a clinical set up.

## Abbreviations

ALP: Alkaline phosphatase
ALT: Alanine aminotransferase
APOP: Advanced protein oxidation product
AR: Arbutin
AST: Aspartate aminotransferease
BA: Benzoic acid
CA: Caffeic acid
CAT: Catalase
CH: (+)-catechin hydrate
EA: Ellagic acid
ECA: (−)-epicatechin
FA: Trans-ferulic acid
GA: Gallic acid
HCHF: High carbohydrate high fat
HPLC-DAD: High performance liquid chromatography- diode array detection
HQ: Hydroquinone
KF: Kaempferol
MC: Myricetin
MDA: Malondialdehyde
NO: Nitric oxide
PCA: *p*-coumaric acid
QU: Quercetin
RA: Rosmarinic acid
RH: Rutin hydrate
SA: Syringic acid
SOD: Superoxide dismutase
TBA: Thiobarbituric acid
TC: Total cholesterol
TCA: Trans-cinnamic acid
VA: Vanillic acid
VL: Vanillin

## References

[1] Novgorodtseva TP, Karaman YK, Zhukova NV, Lobanova EG, Antonyuk MV, Kantur TA (2011). Composition of fatty acids in plasma and erythrocytes and eicosanoids level in patients with metabolic syndrome. Lipids Health Dis.

[2] Daniels SR (2006). The consequences of childhood overweight and obesity. Futur Child.

[3] Swinburn B, Sacks G, Ravussin E (2009). Increased food energy supply is more than sufficient to explain the US epidemic of obesity. Am J Clin Nutr.

[4] Borén J, Taskinen MR, Olofsson SO, Levin M (2013). Ectopic lipid storage and insulin resistance: a harmful relationship. J Internal Med.

[5] Perry RJ, Samuel VT, Petersen KF, Shulman GI (2014). The role of hepatic lipids in hepatic insulin resistance and type 2 diabetes. Nature.

[6] Donnelly KL, Smith CI, Schwarzenberg SJ, Jessurun J, Boldt MD, Parks EJ (2005). Sources of fatty acids stored in liver and secreted via lipoproteins in patients with nonalcoholic fatty liver disease. J Clin Invest.

[7] Scicchitano P, Cameli M, Maiello M, Modesti PA, Muiesan ML, Novo S, Palmiero P, Saba PS, Pedrinelli R, Ciccone MM (2014). Nutraceuticals and dyslipidaemia: beyond the common therapeutics. J Funct Foods.

[8] Kukongviriyapan U, Sompamit K, Pannangpetch P, Kukongviriyapan V, Donpunha W (2012). Preventive and therapeutic effects of quercetin on lipopolysaccharide-induced oxidative stress and vascular dysfunction in mice. Can J Physiol Pharmacol.

[9] Chen Q, Wang E, Ma L, Zhai P (2012). Dietary resveratrol increases the expression of hepatic 7alpha-hydroxylase and ameliorates hypercholesterolemia in high-fat fed C57BL/6J mice. Lipids Health Dis.

[10] Hanhineva K, Törrönen R, Bondia-Pons I, Pekkinen J, Kolehmainen M, Mykkänen H, Poutanen K (2010). Impact of dietary polyphenols on carbohydrate metabolism. Int J Mol Sci.

[11] Kuate D, Kengne APN, Biapa CPN, Azantsa BGK, Wan Muda WAMB (2015). Tetrapleura tetraptera spice attenuates high-carbohydrate, high-fat diet-induced obese and type 2 diabetic rats with metabolic syndrome features. Lipids Health Dis.

[12] Bhaswant M, Poudyal H, Mathai ML, Ward LC, Mouatt P, Brown L (2015). Green and black cardamom in a diet-induced rat model of metabolic syndrome. Nutrients.

[13] Naveed R, Hussain I, Tawab A, Tariq M, Rahman M, Hameed S, Mahmood MS, Siddique AB, Iqbal M (2013). Antimicrobial activity of the bioactive components of essential oils from Pakistani spices against salmonella and other multi-drug resistant bacteria. BMC Complement Altern Med.

[14] Eikani MH, Golmohammad F, Amoli HS, Sadr ZB (2013). An experimental design approach for pressurized liquid extraction from cardamom seeds. Sep Sci Technol.

[15] Aggarwal BB, Prasad S, Reuter S, Kannappan R, Yadev VR, Park B, Kim JH, Gupta SC, Phromnoi K, Sundaram C (2011). Identification of novel anti-inflammatory agents from Ayurvedic medicine for prevention of chronic diseases:“reverse pharmacology” and “bedside to bench” approach. Curr Drug Targets.

[16] Majdalawieh AF, Carr RI (2010). In vitro investigation of the potential immunomodulatory and anti-cancer activities of black pepper (*Piper nigrum*) and cardamom (Elettaria Cardamomum). J Med Food.

[17] Sengupta A, Bhattachanjee S, Aggarwal B, Kunnumakkara A (2009). Cardamom (*Elettaria cardamomum*) and its active constituent, 1, 8-cineole.

[18] Bird RP (1995). Role of aberrant crypt foci in understanding the pathogenesis of colon cancer. Cancer Lett.

[19] Jahan IA, Hossain H, Akbar PN, Rahman MM, Khan TA, Rahman SE, Siraj MA (2014). Antioxidant properties and HPLC assay of bioactive polyphenols of the ethanol extract of *Excoecaria agallocha* stem bark growing in Bangladesh. Brit J Pharm Res.

[20] Niehaus WG, Samuelsson B (1968). Formation of malonaldehyde from phospholipid arachidonate during microsomal lipid peroxidation. Eur J Biochem.

[21] Tracey WR, Tse J, Carter G (1995). Lipopolysaccharide-induced changes in plasma nitrite and nitrate concentrations in rats and mice: pharmacological evaluation of nitric oxide synthase inhibitors. J Pharmacol Exp Ther.

[22] Witko-Sarsat V, Friedlander M, Capeillere-Blandin C, Nguyen-Khoa T, Nguyen AT, Zingraff J, Jungers P, Descamps-Latscha B (1996). Advanced oxidation protein products as a novel marker of oxidative stress in uremia. Kidney Int.

[23] Tiwari BK, Kumar D, Abidi AB, Rizvi SI (2014). Efficacy of composite extract from leaves and fruits of medicinal plants used in traditional diabetic therapy against oxidative stress in alloxan-induced diabetic rats. ISRN Pharmacol.

[24] Khan RA (2012). Protective effects of *Sonchus asper* (L.) hill, (Asteraceae) against CCl(4-)induced oxidative stress in the thyroid tissue of rats. BMC Complement Altern Med.

[25] Misra HP, Fridovich I (1972). The role of superoxide anion in the autoxidation of epinephrine and a simple assay for superoxide dismutase. J Biol Chem.

[26] Jollow DJ, Mitchell JR, Zampaglione N, Gillette JR (1974). Bromobenzene-induced liver necrosis. Protective role of glutathione and evidence for 3,4-Bromobenzene oxide as the Hepatotoxic metabolite. Pharmacol.

[27] Bradley PP, Priebat DA, Christensen RD, Rothstein G (1982). Measurement of cutaneous inflammation: estimation of neutrophil content with an enzyme marker. J Invest Dermatol.

[28] Fabbrini E, Sullivan S, Klein S (2010). Obesity and nonalcoholic fatty liver disease: biochemical, metabolic, and clinical implications. Hepatology.

[29] Adams LA, Lymp JF, Sauver JS, Sanderson SO, Lindor KD, Feldstein A, Angulo P (2005). The natural history of nonalcoholic fatty liver disease: a population-based cohort study. Gastroenterol.

[30] Jung JY, Lim Y, Moon MS, Kim JY, Kwon O (2011). Onion peel extracts ameliorate hyperglycemia and insulin resistance in high fat diet/streptozotocin-induced diabetic rats. Nutrition & Metabolism.

[31] Alam MA, Kauter K, Brown L (2013). Naringin improves diet-induced cardiovascular dysfunction and obesity in high carbohydrate, high fat diet-fed rats. Nutrients.

[32] Furukawa S, Fujita T, Shimabukuro M, Iwaki M, Yamada Y, Nakajima Y, Nakayama O, Makishima M, Matsuda M, Shimomura I (2004). Increased oxidative stress in obesity and its impact on metabolic syndrome. J Clin Invest.

[33] Maddux BA, See W, Lawrence JC, Goldfine AL, Goldfine ID, Evans JL (2001). Protection against oxidative stress-induced insulin resistance in rat L6 muscle cells by mircomolar concentrations of alpha-lipoic acid. Diabetes.

[34] Rudich A, Tirosh A, Potashnik R, Hemi R, Kanety H, Bashan N (1998). Prolonged oxidative stress impairs insulin-induced GLUT4 translocation in 3T3-L1 adipocytes. Diabetes.

[35] Aroor AR, DeMarco VG. Oxidative stress and obesity: the chicken or the egg? Diabetes. 2014;63(7):2216–8. 10.2337/db14-0424 24962921

[36] Azimi P, Ghiasvand R, Feizi A, Hariri M, Abbasi B. Effects of cinnamon, cardamom, saffron, and ginger consumption on markers of glycemic control, lipid profile, oxidative stress, and inflammation in type 2 diabetes patients. Rev Diabet Stud. 2014;11(3-4):258–66. 10.1900/RDS.2014.11.258 PMC5397291 26177486

[37] Nitasha Bhat GM, Nayak N, Vinodraj K, Chandralekha N, Mathai P, Cherian J (2015). Comparison of the efficacy of cardamom (Elettaria Cardamomum) with pioglitazone on dexamethasone-induced hepatic steatosis, dyslipidemia, and hyperglycemia in albino rats. J Adv Pharm Technol Res.

[38] Panchal SK, Poudyal H, Iyer A, Nazer R, Alam A, Diwan V, Kauter K, Sernia C, Campbell F, Ward L (2011). High-carbohydrate high-fat diet-induced metabolic syndrome and cardiovascular remodeling in rats. J Cardiovasc Pharmacol.

[39] Paschos P, Paletas K (2009). Non alcoholic fatty liver disease and metabolic syndrome. Hippokratia.

[40] Parmar M, Shah P, Thakkar V, Gandhi T (2009). Hepatoprotective activity of Amomum Subulatum Roxb against ethanol-induced liver damage. Int J Green Pharm.

[41] Morita T, Jinno K, Kawagishi H, Arimoto Y, Suganuma H, Inakuma T, Sugiyama K (2003). Hepatoprotective effect of myristicin from nutmeg (Myristica Fragrans) on lipopolysaccharide/d-galactosamine-induced liver injury. J Agri Food Chem.

[42] Feillet-Coudray C, Sutra T, Fouret G, Ramos J, Wrutniak-Cabello C, Cabello G, Cristol JP, Coudray C (2009). Oxidative stress in rats fed a high-fat high-sucrose diet and preventive effect of polyphenols: involvement of mitochondrial and NAD(P)H oxidase systems. Free Radic Biol Med.

[43] Noeman SA, Hamooda HE, Baalash AA (2011). Biochemical study of oxidative stress markers in the liver, kidney and heart of high fat diet induced obesity in rats. Diabetol Metab Syndr.

[44] Day CP, James OF (1998). Steatohepatitis: a tale of two "hits"?. Gastroenterol.

[45] Eccleston HB, Andringa KK, Betancourt AM, King AL, Mantena SK, Swain TM, Tinsley HN, Nolte RN, Nagy TR, Abrams GA, Bailey SM (2011). Chronic exposure to a high-fat diet induces hepatic steatosis, impairs nitric oxide bioavailability, and modifies the mitochondrial proteome in mice. Antioxid Redox Signal.

[46] Bouderba S, Sanz MN, Sanchez-Martin C, El-Mir MY, Villanueva GR, Detaille D, Koceir EA (2012). Hepatic mitochondrial alterations and increased oxidative stress in nutritional diabetes-prone Psammomys Obesus model. Exp Diabetes Res.

[47] Lee UE, Friedman SL (2011). Mechanisms of hepatic Fibrogenesis. Best Prac Res Clin Gastroenterol.

[48] Beljaars L, Schippers M, Reker-Smit C, Martinez FO, Helming L, Poelstra K, Melgert BN (2014). Hepatic localization of macrophage phenotypes during Fibrogenesis and resolution of fibrosis in mice and humans. Frontiers Immunol.

[49] Elsegood CL, Chan CW, Degli-Esposti MA, Wikstrom ME, Domenichini A, Lazarus K, van Rooijen N, Ganss R, Olynyk JK, Yeoh GCT (2015). Kupffer cell–monocyte communication is essential for initiating murine liver progenitor cell–mediated liver regeneration. Hepatol.

[50] Brewer M (2011). Natural antioxidants: sources, compounds, mechanisms of action, and potential applications. Compr Rev Food Sci Food Saf.

